# Surgical Treatment Options for Epiglottic Collapse in Adult Obstructive Sleep Apnoea: A Systematic Review

**DOI:** 10.3390/life12111845

**Published:** 2022-11-11

**Authors:** Kyriaki Vallianou, Konstantinos Chaidas

**Affiliations:** 1 Ear, Nose, and Throat (ENT) Department, University Hospital of Larissa, 41334 Larissa, Greece; 2 Ear, Nose, and Throat (ENT) Department, University Hospital of Alexandroupolis, Department of Medicine, Democritus University of Thrace, 68100 Alexandroupolis, Greece

**Keywords:** epiglottis, epiglottic collapse, obstructive sleep apnoea, surgical treatment, surgery

## Abstract

The critical role of epiglottis in airway narrowing contributing to obstructive sleep apnoea (OSA) and continuous positive airway pressure (CPAP) intolerance has recently been revealed. This systematic review was conducted to evaluate available surgical treatment options for epiglottic collapse in adult patients with OSA. The Pubmed and Scopus databases were searched for relevant articles up to and including March 2022 and sixteen studies were selected. Overall, six different surgical techniques were described, including partial epiglottectomy, epiglottis stiffening operation, glossoepiglottopexy, supraglottoplasty, transoral robotic surgery, maxillomandibular advancement and hypoglossal nerve stimulation. All surgical methods were reported to be safe and effective in managing selected OSA patients with airway narrowing at the level of epiglottis. The surgical management of epiglottic collapse can improve OSA severity or even cure OSA, but can also improve CPAP compliance. The selection of the appropriate surgical technique should be part of an individualised, patient-specific therapeutic approach. However, there are not enough data to make definitive conclusions and additional high-quality studies are required.

## 1. Introduction

Obstructive sleep apnoea (OSA) is a common sleep disorder characterised by repeated episodes of partial or complete airway collapse at various levels of the upper respiratory tract during sleep, which leads to a decrease or cessation of airflow [1]. OSA affects at least 7.8% of the adult population, with prevalence exceeding 50% in some countries [2], and is characterised by oxygen desaturation, sleep fragmentation and excessive daytime sleepiness [3]. Untreated OSA is an important risk factor of cardiovascular disease, arterial and pulmonary hypertension, arrythmias, diabetes and mortality [4]. 

The gold-standard method for OSA diagnosis is overnight polysomnography [3,5]. Airway obstruction can be single- or multi-level, including the velum, the oropharynx, the tongue base and/or the epiglottis. Epiglottic collapse was reported to concern a relatively small number of OSA patients and is often overlooked [6]. However, the actual prevalence seems to be higher, as the diagnosis of epiglottic collapse can elude doctors and be underestimated on awake endoscopy [7]. The addition of drug-induced sedation endoscopy (DISE) to the diagnostic approach helps surgeons in identifying the level of obstruction and provides the ability for more targeted treatment, increasing its success rate [8,9].

Continuous positive airway pressure (CPAP) is the first-line therapy in OSA, but is often associated with poor compliance [9]. Among alternative therapeutic modalities, surgical treatment constitutes an effective option in selected patients and especially in those with narrowing at the level of the epiglottis [10,11]. The aim of this systematic review is to provide an overview of the current literature regarding surgical treatment options in OSA patients with epiglottic collapse.

## 2. Materials and Methods

The Pubmed and Scopus databases were searched for relevant journal articles up to and including March 2022. Search terms included ‘sleep apnoea’, ‘epiglottic collapse’ or ‘epiglottis’ or ‘epiglottic obstruction’, ‘treatment’ or ‘management’ or ‘therapy’. We aimed to identify all full-text articles that examined the surgical treatment options for treating epiglottic collapse in patients with obstructive sleep apnoea. The eligibility criterion for inclusion in the review was a specific focus on the surgical treatment of epiglottic collapse in an adult population (>16 years of age). Studies with no data on surgical management, articles concerning paediatric patients (≤16 years old), reviews and books were excluded. Non-English articles and animal and model studies were also excluded. Two authors independently performed the article search, article selection and data extraction. The reference lists of the chosen articles were manually searched to further identify relevant articles. The PRISMA guidelines were adapted for the current review. 

## 3. Results

### 3.1. Search Results and Article Selection

**Figure 1:**
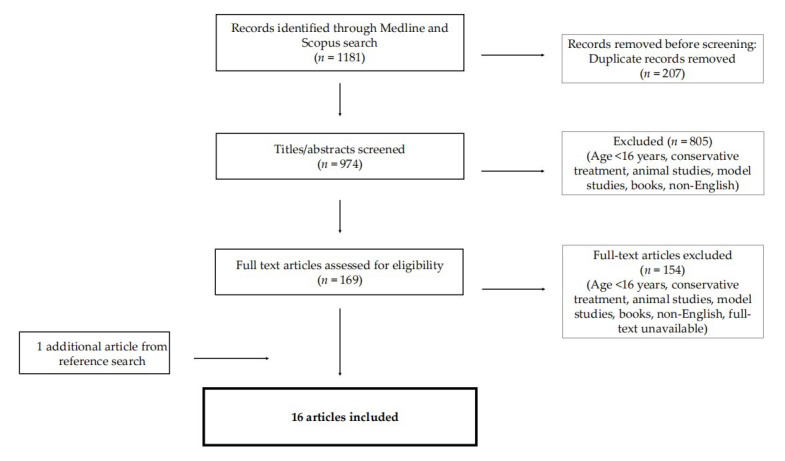
Literature search and article selection; *n*: number of studies.

The electronic database search identified 1181 articles. After the removal of 207 duplicates, the articles were screened by evaluating the titles and abstracts and selection was made based on the appliance of inclusion and exclusion criteria. A total of 169 articles were selected and full texts were retrieved. After further evaluation of their full text, 154 articles were excluded for the following reasons: language restrictions, full text unavailable, conservative treatment of epiglottic collapse, paediatric patients, animal studies, anatomic location of airway obstruction, books and reviews. In total, sixteen studies met the eligibility criteria, as one additional article was added from the reference search (Figure 1). The articles were categorised into three case reports (CRs), four case series (CSs), seven retrospective cohort studies (RCSs), and two prospective cohort studies (PCSs). 

The variations between the studies with regard to study type, patient characteristics and type of surgery are outlined in Table 1. The quality of each study was evaluated by using the GRADE (Grading of Recommendations Assessment, Development and Evaluation) system [12]. Additionally, Table 2 shows the preoperative and postoperative outcomes, including the Oxygen Desaturation Index (ODI), the Apnoea Hypopnoea Index (AHI) and the Epworth Sleepiness Scale (ESS) scores for each study, in order to evaluate the effectiveness of surgical treatment on epiglottic collapse.

### 3.2. Partial Epiglottectomy 

A total of three studies were identified that reported CO_2_ partial epiglottis resection for the treatment of epiglottic collapse in OSA patients [14,18,27]. In a study published by Catalfumo et al., twelve patients with epiglottis collapse underwent partial epiglottectomy, with no serious complications. The results indicate that this operation can increase the OSA treatment success rate by 10–15%, with a significant improvement in oxygen blood saturation, apnoea duration and the apnoea hypopnea index (AHI) score after surgery [27]. Golz et al. performed U-shaped partial epiglottectomy with a CO_2_ laser in 27 adults with OSA and laryngomalacia, uneventfully. Postoperative sleep studies demonstrated a statistically significant improvement in 85% of patients and complete relief of their respiratory symptoms. A significant decrease in the respiratory disturbance index (RDI) was achieved in 77.8% of the patients [18]. Verse et al. [14] published a case report of a 70-year-old man who underwent a partial epiglottectomy using the CO_2_ laser, due to a very large epiglottis. After the surgery, the patient regained the ability to sleep in the supine position with a reduced AHI score and complete disappearance of all respiratory disturbances [14]. Not only the CO_2_ laser, but also monopolar diathermy can be used for partial epiglottectomy [15,23]. Oluwasamni et al. reported four cases undergoing endoscopic partial epiglottectomy using monopolar diathermy to treat a floppy epiglottis. This was found to be safe and effective with minimal morbidity [15]. Similarly, in a study published by Jeong et al., the authors used monopolar electrocautery to proceed to partial resection of the epiglottis, in order to improve CPAP usage. The patients were relieved from the feeling of suffocation with a reduction in the AHI score and satisfactory use of CPAP [23]. 

### 3.3. Epiglottis Stiffening Operation

The literature search revealed two articles that described the epiglottis stiffening operation (ESO) as a therapeutic approach to epiglottic collapse in OSA patients [21,26]. Salamanca et al. [26] used suction cautery to cauterise the lower half of the lingual surface of the epiglottis in the area between the lateral glosso-epiglottic folds to induce stiffening and scar retraction of tissues as a result of secondary healing. The authors highlighted the importance of reaching the perichondrium of the lingual side of the epiglottis, to induce stiffening in the direction of median thyroepiglottic ligament. ESO was performed in 14 patients without complications such as dysphagia or aspiration [26]. Leone et al. published a retrospective study, in which one patient developed a sleep-related breathing disorder, due to a floppy epiglottis as a consequence of chemo- and radiotherapy for head and neck cancer. The patient underwent an epiglottis stiffening operation without postoperative complications and reached a resolution of OSA with normalisation of the AHI after surgery (from 47.7 episodes/h prior to surgery, the AHI decreased to 4.7 episodes/h, postoperatively) [21].

### 3.4. Glossoepiglottopexy

Roustan et al. developed a surgical technique that provides a support to the epiglottis without destroying its function during swallowing. They analysed a group of 20 patients who underwent glossoepiglottopexy using a CO_2_ laser and pharyngoplasty. There was a significant reduction in the ESS score, AHI and ODI with normal swallowing in all patients, postoperatively [28].

### 3.5. Supraglottoplasty 

Supraglottoplasty with the intraoral and laryngoscopic approach was carried out by Li et al. in one patient with OSA caused by laryngomalacia [17]. There was a good response to treatment with improvement in snoring, daytime sleepiness and sleep apnoea. Postoperative endoscopy revealed a reduction in the size of the epiglottis and arytenoids without collapse of the supraglottic tissue.

### 3.6. Transoral Robotic Surgery (TORS)

Three studies published the results of robotic-assisted surgery in the epiglottis for OSA [20,22,25]. Shehan et al. [25] reported the first robotic-assisted epiglottopexy in the adult otolaryngology literature. Namely, the authors described two cases with epiglottic collapse undergoing robotic-assisted epiglottopexy. The first patient showed a decrease in the AHI and ODI and the second patient had a significant decrease in the AHI and ESS [25]. Kayhan et al. [20] evaluated the results of combined multilevel surgery with transoral robotic surgery (TORS) in patients with OSA and multilevel airway obstruction. DISE was performed in all patients in order to identify the level of obstruction and determine the type of surgery required. A total of 24 patients underwent a base of tongue (BOT) reduction and epiglottoplasty. There was no need for tracheostomy in any of the patients. Sleep apnoea was cured in 72% of the patients, whereas an additional 8% of the patients met the criteria for surgical success. Another prospective study published by Arora et al. [22] included 10 patients who underwent TORS for tongue base reduction and epiglottoplasty. A 64% success rate was achieved with a normal postoperative polysomnography in 36% of cases at six months. There was a 51% reduction in the mean AHI score. 

### 3.7. Maxillomandibular Advancement

Our search identified two studies by Liu et al. in which patients with OSA and epiglottic collapse underwent maxillomandibular advancement (MMA) [16,19]. The first retrospective cohort study included four patients with partial or complete epiglottic collapse and a mean preoperative AHI score of 59.8 episodes per hour. Post-MMA, the AHI and ODI scores had a statistically significant reduction, whereas postoperative sleep endoscopy showed an improvement in the collapsibility of the epiglottis [19]. Subsequently, Liu et al. published a retrospective cohort study of 20 OSA patients undergoing MMA. A total of 6 out of 20 patients were found to have narrowing at the level of the epiglottis at the preoperative assessment, which was resolved in half (three) of them after surgery. In contrast, three patients had a residual epiglottic collapse despite surgery. The authors found that MMA increases the stability of the lateral pharyngeal wall, followed by the velum and the tongue base. The results were assessed with DISE and computational fluid dynamics [16].

### 3.8. Hypoglossal Nerve Stimulation 

A retrospective cohort study by Xiao et al. included 13 patients who underwent hypoglossal nerve stimulation (HNS) as a treatment for partial or complete epiglottic collapse and obstructive sleep apnoea. The authors reported a significant improvement in the AHI, ODI and ESS three months after HNS implantation [24]. In a study by Heiser et al., a 64-year-old male presented with residual OSA with an increased AHI and evidence of a floppy epiglottis six months after upper airway stimulation [13]. A change of the electrode configuration for stimulation from bipolar to monopolar produced a clear opening of the epiglottis and the patient improved significantly.

## 4. Discussion

According to the guidelines published by the American Academy of Sleep Medicine, a referral to a sleep surgeon should be considered for patients with OSA and a BMI of less than 40 kg/m^2^ who are intolerant or unaccepting of CPAP [29]. In adult OSA, airway obstruction is often present at multiple levels and a thorough assessment of the upper respiratory tract is necessary. DISE provides valuable information about the presence, level and type of obstruction and can guide the selection of the appropriate surgical method, increasing its success rate [9]. The role of DISE is crucial, especially in the presence of epiglottic collapse, which is usually difficult to identify on awake endoscopy. Although the role of the epiglottis in OSA was underestimated for many years, several recent reports confirm the increased prevalence of this type of obstruction and highlight the importance of targeted management in order to resolve OSA or improve CPAP compliance [7]. Our review demonstrates that various surgical methods with satisfactory results and minimum morbidity exist. The selection of the optimal surgical option should primarily be guided by the type of underlying pathology, although other factors, such as patient characteristics and preference, and equipment availability, should also be considered.

The type of epiglottic collapse can be divided into anterioposterior, which is the most common, and lateral collapse [30]. Before the advent of DISE, epiglottic collapse evaluated by an awake clinical examination was reported to occur in 11.4% of OSA patients [28], but its prevalence has increased since the wider use of DISE [30]. A study by Fernandez-Julian et al. found that the epiglottis was involved in the obstruction of 24.1–28.4% of the patients according to an awake examination, a rate that increased to 36.4% based on DISE [31]. Lan et al. [32] noted anterioposterior or lateral epiglottic collapse in 42.2% of their patients. Another study using DISE in 324 patients reported a floppy epiglottis in 18.5% of them [33]. In most cases, epiglottic collapse co-exists with obstruction at other sites, whereas isolated epiglottic collapse is seen in a significantly smaller number of patients, with a rate ranging between 3.5% and 14.4% [18,34,35]. The prevalence of epiglottic collapse is significantly higher when evaluating OSA patients who have previously failed upper airway surgery, ranging from 44–72.9% [36,37]. 

The pathophysiology of epiglottic collapse remains not well understood and several mechanisms have been proposed to explain this phenomenon. Epiglottic collapse may occur secondary to anterioposterior collapse of the base of the tongue, pushing the epiglottis backwards, or due to underdevelopment of the epiglottis, which leads to lateral collapse. An underdeveloped epiglottis can cause laryngomalacia and sleep apnoea. Another described mechanism is the complete isolated anterioposterior epiglottic collapse occurring during inspiration, also known as the ‘trapdoor phenomenon’ [33]. In this case, the epiglottis prolapses into the posterior pharyngeal wall during inspiration, causing airway obstruction. Moreover, a previous history of radiotherapy for oropharyngeal or laryngeal cancer has been reported to increase the risk for OSA by causing oedema and malacia of the epiglottis and/or a floppy epiglottis [38].

The relationship between the shape of the epiglottis and its potential collapse is still controversial. Sung et al. assessed 11 cases with isolated epiglottic collapse and 44 controls and found no differences in terms of epiglottic shape or curvature between the two groups [35]. Another study showed no significant correlation between the position of the epiglottis and the presence of collapse [33]. In contrast, Kanemaru et al. identified a positive association between a concave posterior surface of the epiglottis and the degree of airway collapse and, thus, the severity of OSA [39]. Catalfumo et al. [27] compared the results of full polysomnography studies and found a correlation between the severity of OSA and the position of the epiglottis. Kuo et al. [40] pointed out the connection between the epiglottic length and epiglottic collapse, which can be screened with a CT scan or cephalometry. An epiglottic length of more than 1.66 cm was indicative of collapse of the epiglottis. Additionally, high hyoid mobility was correlated with epiglottic collapse in OSA patients. In contrast, the epiglottic angle did not seem to play a role [40]. 

Satisfactory management in OSA patients presupposes the identification of the location of the upper airway obstruction. Several diagnostic methods have been used to assess upper airway patency and identify associated pathology in patients with OSA, including awake flexible endoscopy, computed tomography (CT), magnetic resonance imaging (MRI), cephalometry, intrapharyngeal pressure manometry and DISE. Since its first introduction in 1991, DISE has gained popularity, as it allows a direct dynamic evaluation of the upper airway during drug-induced “sleep” [6]. This technique is a useful method of assessing the location, severity and pattern of airway obstructions. To better describe DISE findings in a standardised way, the VOTE (velum, oropharynx, tongue base, epiglottis) classification has been widely used with very good intra- and inter-rater reliability [8]. All patients should undergo an awake fibre-optic nasolaryngoscopy prior to any surgical procedure to enable the surgeon to fully assess the upper airway and identify any additional pathology. Those with a high suspicion of epiglottic collapse should also undergo DISE, as it is challenging to fully assess this type of obstruction on awake endoscopy [37].

Although CPAP is the first-line therapy in OSA, its efficacy seems to be limited in patients with epiglottic collapse [35]. The positive airway pressure can push the epiglottis downward into the laryngeal inlet, leading to significant narrowing of the upper airway, worsening of OSA and/or CPAP intolerance [30]. A recent study by Sung et al. suggests that patients with epiglottic collapse have a higher CPAP adherence failure rate than patients without epiglottic collapse [41]. Salamanca et al. and Shehan et al. also agree that a poor response to CPAP in patients with epiglottic collapse occurs because the positive pressure may worsen the epiglottic obstruction [25,26]. A collapsing epiglottis has been found in 15–31.4% of adult patients with OSA who did not tolerate CPAP therapy or in whom CPAP treatment was ineffective [28,42,43,44]. Kim et al. confirmed this assertion by publishing an article with an OSA patient with worsening epiglottic collapse during CPAP application in a video presentation [45]. In case of residual obstruction after CPAP, an upper airway reassessment should be performed to determine the presence and type of epiglottic collapse [46], as these patients require different management. 

Positional therapy and mandibular advancement devices are among the non-surgical treatment options that have been suggested to manage epiglottic collapse. Both treatment modalities seem to improve airway patency in those patients but only in mild cases, as in the presence of severe OSA, additional treatment is usually required [45,47].

In contrast to conservative management, surgical therapy of epiglottic collapse seems to be more effective. Several surgical techniques aiming to resolve epiglottic narrowing have been reported in the literature with overall good results. The available operations include less invasive techniques, such as partial epiglottectomy, glossoepiglottopexy and supraglottoplasty, and relatively more aggressive techniques, including transoral robotic surgery, maxillomandibular advancement and hypoglossal nerve stimulation. However, the use of new technologies, such as diathermy and CO_2_ and thulium lasers, along with the gradual increase in surgical experience, have improved the safety of the operations [30].

It is known that the epiglottis participates in swallowing and is also involved in preventing food aspiration by closing the laryngeal aditus during swallowing. Although the main objective of surgical management is the improvement of upper airway patency by resolving epiglottic collapse, the function of the epiglottis should also be preserved.

Partial epiglottectomy is an effective tool for patients with laryngomalacia or trapdoor epiglottis. A carbon dioxide laser and monopolar diathermy have both been cited to be useful in the surgical treatment of OSA caused by laryngomalacia [15,27]. Partial epiglottectomy with a CO_2_ laser has also been used to excise the redundant mucosa of the arytenoids. This instrument provides a high degree of precision, while maintaining homeostasis and minimising postoperative oedema. The technique was found to be safe without complications. By cutting out the upper-middle one third to half of the epiglottis, the aerodynamic shape of the hypopharynx is changed, resolving the obstruction caused by the epiglottis. 

It is often challenging to determine the optimal volume of effective epiglottic resection without postoperative complications, as excessive epiglottic resection can cause aspiration, whereas insufficient resection carries the risk of residual obstruction. Some studies suggest leaving a residual 3–4 mm rim of healthy mucosa along the entire profile of the epiglottis [14,27,40]. Bartolomeo et al. [48] report that V-shaped partial epiglottectomy minimises the risk of aspiration, while ensuring satisfactory airflow through the epiglottic V during epiglottic movement.

The epiglottis stiffening operation (ESO) was first described by Salamanca et al. [26] in 2019. Following this technique, stiffening and scar retraction leads to flexion of the epiglottis towards the tongue base by secondary intention. The authors suggested leaving some healthy tissue along the free border of the epiglottis to allow the activation of reflexes. Overall, the ESO was found to be a safe and effective procedure with a shorter healing time than partial epiglottectomy [26]. 

Another minimally invasive technique which can achieve resolution of epiglottic collapse, while preserving the function of the epiglottis, is glossoepiglottopexy. Transoral glossoepiglottopexy constitutes a safe and effective surgical treatment option for adults with OSA and epiglottic collapse and is associated with a lower risk of complications compared to partial epiglottectomy [27,28]. This method provides stable support to the epiglottis, protects its function during swallowing and creates a barrier for posterior falling off the tongue base by reinforcing the wall of the airway [28].

Transoral robotic surgery (TORS) was first reported in 2010 as a modification of open tongue base reduction and hyoid epiglottopexy to treat OSA [49]. It has been demonstrated that TORS of the tongue base with or without epiglottoplasty by using a CO_2_ or thulium laser is a safe and effective alternative treatment option for selected patients, when other treatment options failed. The ideal candidates are patients with an obstruction at the level of tongue base and/or epiglottis and the procedure can be completed without major complications or the need for tracheostomy or open surgery [22]. Although the surgeon has no haptic feedback intra-operatively, TORS provides an excellent approach at the hypopharynx with three-dimensional visualisation of the surgical field [20]. 

Maxillomandibular advancement constitutes an alternative surgical treatment for patients with an intolerance to CPAP therapy, particularly for patients with severe lateral pharyngeal and epiglottic collapse diagnosed on DISE. MMA is considered one of the most effective treatment options for patients with OSA, but is associated with significantly higher complication rates compared to other surgical options [50].

Non-obese patients with a history of previous CPAP failure or intolerance should also be screened for upper airway stimulation [13,24]. Stimulation of the hypoglossal nerve and activation of the genioglossus muscle unbars the base of the tongue and the soft palate. Additional stimulation settings, such as changing the configuration of the stimulation electrode, may optimise muscle recruitment to favour upper airway dilation at the levels of the soft palate, tongue and epiglottis [13]. Despite the relatively excessive surgical dissection required for implantation, hypoglossal nerve stimulation is a safe and effective method with promising outcomes and low associated morbidity [24].

The aim of this systematic review was to assess the efficacy of surgical therapy in patients with OSA and epiglottic collapse. The current evidence shows that several surgical options with satisfactory outcomes and safety profiles exist. However, this review carries out certain limitations and conclusions should be made with caution. First, a systematic review was conducted but not a meta-analysis. The studies included were all either retrospective or prospective observational studies. None of the studies was randomised or multi-centre. Additionally, the outcomes were based on small sample sizes. There was also a variation in the methodology and the duration of follow-up assessment after surgery. This literature review included studies specifically mentioning the effect of surgical treatment in patients with OSA and epiglottic collapse. However, it should be taken into consideration that other techniques such as tongue base advancement may also affect airway patency at the level of the epiglottis. Our literature search revealed that most studies have focused on the impact of a surgical technique on the level of the tongue base or on upper airway patency in general, without specifically evaluating and reporting outcomes on epiglottic collapse. These studies are beyond the scope of this review and were excluded. 

## 5. Conclusions

Since DISE has become a popular method for upper airway examination, the critical role of the epiglottis in airway narrowing contributing to OSA has been revealed. Unfortunately, CPAP intolerance or failure is relatively common in patients with epiglottic collapse and, thus, alternative treatment options should be considered. Several surgical techniques have been described in the literature, with overall satisfactory results and safety profiles. The surgical management of epiglottic collapse can improve OSA severity or even cure OSA but can also improve CPAP compliance. Moreover, as upper airway obstruction is often multilevel, epiglottic surgery can also be combined with other upper airway procedures. The selection of the appropriate surgical technique should be made based on the type of airway obstruction, patient characteristics and preferences, surgical skills and equipment availability, as part of an individualised, patient-specific therapeutic approach. Nevertheless, our findings should be evaluated with caution due to the low quality of the included studies. For that reason, there is a need for high-quality randomised trials with large sample sizes to allow us to safely determine the effect of surgery in patients with OSA and epiglottic collapse.

## Figures and Tables

**Table 1:** Individual study characteristics.

Study	Study Type	Patients Nr	Sex	Age (Years)	BMI (kg/m^2^)	Physical Examination	Surgical Procedure	Follow-Up	Study Quality (GRADE)
Heiser [13]	CR	1	Male	64	25.9		Hypoglossal nerve stimulation	6 months	Very low
Verse [14]	CR	1	Male	70	24.2	Large epiglottis adhered to posterior pharyngeal wall	CO_2_ partial epiglottic resection	7 days	Very low
Oluwasamni [15]	CS	4	Male	50–65		Floppy epiglottis	Endoscopic partial epiglottidectomy	2 months–3.5 years	Very low
Liu [16]	RCS	20	17 males/ 3 females	44 ± 12			Maxillomandibular advancement	6 months	Low
Li [17]	CR	1	Male	24		Long epiglottis touching the uvula and tilted posteriorly against the pharyngeal wall	Supraglottoplasty	6 months	Very low
Golz [18]	RCS	27	21 males/ 6 females	19–68	23.4 ± 4.2	Long, lax and flaccid epiglottis collapsing into the laryngeal inlet	CO_2_ partial epiglottectomy	14–52 months (mean:32.3 months)	Low
Liu [19]	RCS	4/16	15 males/1 female	47 ± 10.9	29.4 ± 5.1	Partial collapse (anterioposterior:2, lateral:1), complete anterioposterior:1	Maxillomandibular advancement	6 months	Very low
Kayhan [20]	RCS		19 males/6 females	50.1± 8.5	30.7 ± 5.5		TORS	3 months	Low
Leone [21]	RCS	1/6	Male	58	25.3	Floppy epiglottis, epiglottis malacia	Epiglottis stiffening operation	8 months	Very low
Arora [22]	PCS	10/14	13 males/1 female	54.3 ± 14.6	28.7 ± 2.8	Concurrent epiglottic collapse	TORS	18.9 ± 6.2 months	Low
Jeong [23]	CS	2	Male	Pt1: 50 Pt2: 58	Pt1:29.1 Pt2:25	Complete epiglottic collapse	Partial epiglottectomy	1 year	Very low
Xiao [24]	RCS	13/48	32 males/16 females	57–69 (66)	28.6		Hypoglossal nerve stimulation	3 months	Low
Shehan [25]	CS	2	Male	Pt1: 60 Pt2: 55			Robotic-assisted epiglottopexy	Pt1: 1 year	Low
Salamanca [26]	RCS	14	13 males/1 female	47–76	25.6 (22.1–34)		Epiglottis stiffening operation	3 months	Low
Catalfumo [27]	PCS	12		42.3 ± 14.6		Nine patients: stage 2 epiglottic position (45–90 degrees), Three patients: stage 3 epiglottic position (over 90 degrees)	CO_2_ partial epiglottis resection	1 year	Low
Roustan [28]	CS	20	16 males/4 females	38–63			Transoral glossoepiglottopexy	6 months	Low

Values are given as number, mean ± SD or range; Nr: number, BMI: body mass index, GRADE: grading of recommendations assessment, development and evaluation, CR: case report, CS: case series, RCS: retrospective cohort study, PCS: prospective cohort study, CO_2_: carbon dioxide laser, TORS: transoral robotic surgery, Pt: patient.

**Table 2:** Preoperative and Postoperative Outcomes.

Study	Preoperative ODI (Events/h)	Preoperative AHI (Episodes/h)	Preoperative ESS	Preoperative SaO_2_ (%)	Postoperative ODI (Events/h)	Postoperative AHI (Episodes/h)	Postoperative ESS
Heiser [13]		36.3		Min: 76		20.7	
Verse [14]	11.1	4.8	7	Min: 77	3.8	0.4	
Oluwasamni [15]							
Liu [16]	38.7 ± 30.3	53.6 ± 26.6			8.1 ± 9.2	9.5 ± 7.4	
Li [17]	21		9	Min: 89.1	6.9		5
Golz [18]	26–65 (45 ± 14.6)			Min: 58–87 (Mean: 66 ± 17.6)	14 ± 5.1		
Liu [19]	45 ± 29.7	59.8 ± 25.6	19.5 ± 2.9	Min: 80.8 ± 7.6	5.7 ± 4.4	9.3 ± 7.1	7.1 ± 2.6
Kayhan [20]		28.7 ± 17.8	13.5 ± 2.8	Min: 80.7 ± 7.6		9.4 ± 12.4	3.4 ± 1.6
Leone [21]		47.7				4.7	
Arora [22]		35.6 ± 19.7	14.9 ± 5	Mean: 92.9 ± 1.8		21.2 ± 24.6	All patients: normal (<10)
Jeong [23]	Pt1: 46.1	Pt1: 57.8, Pt2: 28.4		Pt1 min: 73, Pt2 min: 69		Pt1: 50.5, Pt2: 25.7	
Xiao [24]							
Shehan [25]		Pt1: 35, Pt2: 28	Pt2: 8			Pt1: 31, Pt2: 6	Pt1: 3, Pt2: 2
Salamanca [26]		0.1–57.6	1–9			0.2–6.8	1–7
Catalfumo [27]		42 ± 16.4		Min: 68 ± 8.6	12 ± 4.6	8 ± 3.2	
Roustan [28]	23 ± 14.3	23.6 ± 6.5	16.5 ± 4.3	Mean: 86.9 ± 2.3		5.2 ± 3.2	3.1 ± 2.5

Values are given as mean ± SD or range; ODI: oxygen desaturation index, h: hour, AHI: apnoea hypopnea index, ESS: Epworth Sleepiness Scale, SaO_2_: oxygen saturation, Min: minimum, Pt: patient.

## Data Availability

Not applicable.
